# Fracture Resistance and Failure Modes of Ultrathin Occlusal Veneers Made of Different Ceramic Materials: An In Vitro Study

**DOI:** 10.34172/joddd.44718

**Published:** 2026-06-30

**Authors:** Mohamed Ezzat Elbanna, Hesham Ibrahim Othman, Ahmed Mohammed Leithy Alameldin

**Affiliations:** ^1^Fixed Prosthodontics Department, Faculty of Dentistry, Sinai University, North Sinai, Egypt; ^2^Fixed Prosthodontics Department, Faculty of Dental Medicine, Al-Azhar University, Cairo, Egypt

**Keywords:** Failure mode, Fracture resistance, Lithium disilicate, Occlusal veneer, Zirconia

## Abstract

**Introduction:**

The current in vitro study was designed to assess the failure mode and fracture resistance of ultrathin occlusal veneers made from different ceramic materials.

**Methods:**

Twenty-four ultrathin occlusal veneers were fabricated on maxillary extracted first premolar teeth. Three ceramic materials were used: lithium disilicate (LDS), zirconia-reinforced lithium disilicate (ZLS), and zirconium dioxide (ZIR). An adhesive luting agent was used to adhesively bond occlusal veneers. Each sample was subjected to thermocycling, ranging from 5 to 55°C. Fracture resistance was tested using a universal testing machine, and fractured surfaces were examined with a stereomicroscope and a scanning electron microscope (SEM) to assess failure modes.

**Results:**

A statistically significant difference was observed; the highest mean fracture resistance was observed in LDS (1741±308.3 N), followed by ZLS (1192±89.44 N), and then ZIR (866.9±60.23 N), which had the lowest mean fracture resistance. The failure mode of the ZIR group differed significantly from those of the other groups.

**Conclusion:**

All the tested ceramic materials used as ultrathin occlusal veneers exhibited fracture loads significantly higher than the average physiological masticatory forces. Compared with the other two groups, LDS had the highest fracture resistance value. ZIR occlusal veneers exhibited more repairable fractures than LDS and ZLS.

## Introduction

 Tooth surface loss is a complex process with multiple etiologies, including chemical (erosion) and mechanical (attrition, abrasion, and abfraction) factors. Occlusal defects may develop when pathological and functional defects, such as extensive tooth wear or decay, damage occlusal enamel, exposing the underlying dentin. Gradual non-carious tooth loss negatively affects chewing ability, overall dental satisfaction, and quality of life. Deterioration of the tooth surface and underlying tissues can affect both function and aesthetics, making treatment decisions more challenging in advanced cases.^1^ Dental erosion, or premature enamel loss, can be induced by acidic substances, gastric reflux, medications, and bulimia nervosa. Erosion occurs on occlusal surfaces of posterior teeth, develops slowly, and is characterized by non-carious lesions.^2,3^

 Conventional approaches to tooth loss management may require full-mouth rehabilitation with full- or partial-coverage restorations. Occlusal veneers and other partial-coverage restorations may be a conservative approach to restoring damaged teeth in such cases.^4^ In adolescent patients, intervention should be as minimally invasive as possible to preserve sound tooth structure. Minimally invasive treatments reduce postoperative dentin sensitivity, preserve tooth structure, and preserve pulp vitality.^5^ Ultrathin occlusal veneers are a highly recommended treatment option for tooth structure loss because of the enhanced mechanical properties of various restorative materials and improved adhesion, which enable the restoration of minimal tooth structure damage in thin sections.^6^

 Recently, computer-aided design and computer-aided manufacturing (CAD/CAM) technology has been extensively used in digital prosthodontics. Since many materials are now produced entirely in industrial environments, high standards that are rarely met in laboratory procedures are guaranteed. CAD/CAM fabrication’s precise quality control greatly reduces the likelihood of faults or flaws in an industrial environment and facilitates evaluation of the blocks’ mechanical strength.^7,8^ There is an ongoing debate over the ideal restorative material for indirect restorations as new materials are introduced to the market.^6^

 Glass ceramics have been recommended as a good option for indirect minimally invasive procedures. However, using glass ceramic for posterior restoration at lower thicknesses has a considerable risk of technical challenges. Numerous ceramic materials with diverse mechanical properties have been introduced to the dental market, including zirconia and lithium disilicate ceramics.^9^ The use of glass ceramic and oxide ceramic materials in full- or partial-coverage restorations and fixed partial dentures has demonstrated promising outcomes in terms of survival rate, integrity, wear resistance, and color stability.^10^ Zirconia has been added to lithium disilicate to strengthen it and combine the best characteristics of both materials. Pressable and machinable, zirconia-reinforced lithium silicate ceramics have dense crystalline substructures that offer good mechanical and optical qualities.^11^

 Ceramic fracture is considered a primary factor in restoration failure.^12^ In previous research, occlusal veneers of various thicknesses and materials have been evaluated for fracture resistance, but the results have not always been consistent. Further investigation is needed to identify the most suitable material for occlusal veneers. It is important to know why a tooth or dental restoration fails. There are various ways a tooth or restoration can fail, with fractures among the most frequent clinical issues. This may directly influence the choice of whether to extract or repair the damaged tooth.^13^

 Failure mode analysis is a technique that aids in determining the origin, direction, and cause of fracture. However, a lack of knowledge about the types, behaviors, and varieties of tooth structural fractures could lead to misdiagnosis and improper treatment.^13^ The American Association of Endodontics (AAE) has established five categories for longitudinal fractures to provide academics with universal definitions. According to this classification, craze lines are visible only on enamel. Fractured cusps, split teeth, and cracks affect the dentin, enamel, and occasionally even the pulp or root by extending apically from the occlusal surface.^14^ In previous studies, the fracture resistance of ultrathin occlusal veneers with various materials has been compared, with no consistent conclusion. More research is needed to determine the most suitable material for occlusal veneers.^15^

 The aim of this study was to assess the fracture resistance and failure modes of ultrathin occlusal veneers fabricated from different ceramic materials. The first null hypothesis stated that different restorative materials would not affect the fracture resistance of ultrathin occlusal veneers. The second null hypothesis was that the mode of failure was the same for various materials.

## Methods

 Twenty-four sound, extracted maxillary premolars with comparable mesiodistal and buccolingual dimensions, recently extracted for orthodontic reasons, were collected. To standardize the tooth dimensions, a 0.3-mm range was permitted for each measurement. To detect dental caries, fractures, cracks, or signs of resorption, teeth were examined under magnification with a stereomicroscope (Nikon Japan, SMZ745T, USA). An ultrasonic scaler (UDS-A LED, Guilin Woodpecker CO, Ltd., Guangxi, 541004, PR China) was used to remove any debris from the teeth. After 10 minutes of disinfection with a sodium hypochlorite solution, the teeth were stored in distilled water at 37ºC.

 A parallometer was used to mount the teeth in a cylindrical PVC ring with self-curing acrylic resin (Acrostone, Cold cure, ISO13485, England) (Figure 1a). To create a layer approximately 0.2‒0.3-mm thick, a uniform coating of dental wax (Tauxhwachs, Dipping wax, BEGO, Germany) was applied to the embedded region of the tooth roots 2 mm below the cementoenamel junction. To replicate the periodontal ligament membrane, silicone light body impression material (Elite HD+, ISO, Zhermack Spa, Italy) was injected around the roots after the acrylic resin had completely set and the wax had been removed. Then, excess impression material was removed using a scalpel.

**Figure 1:**
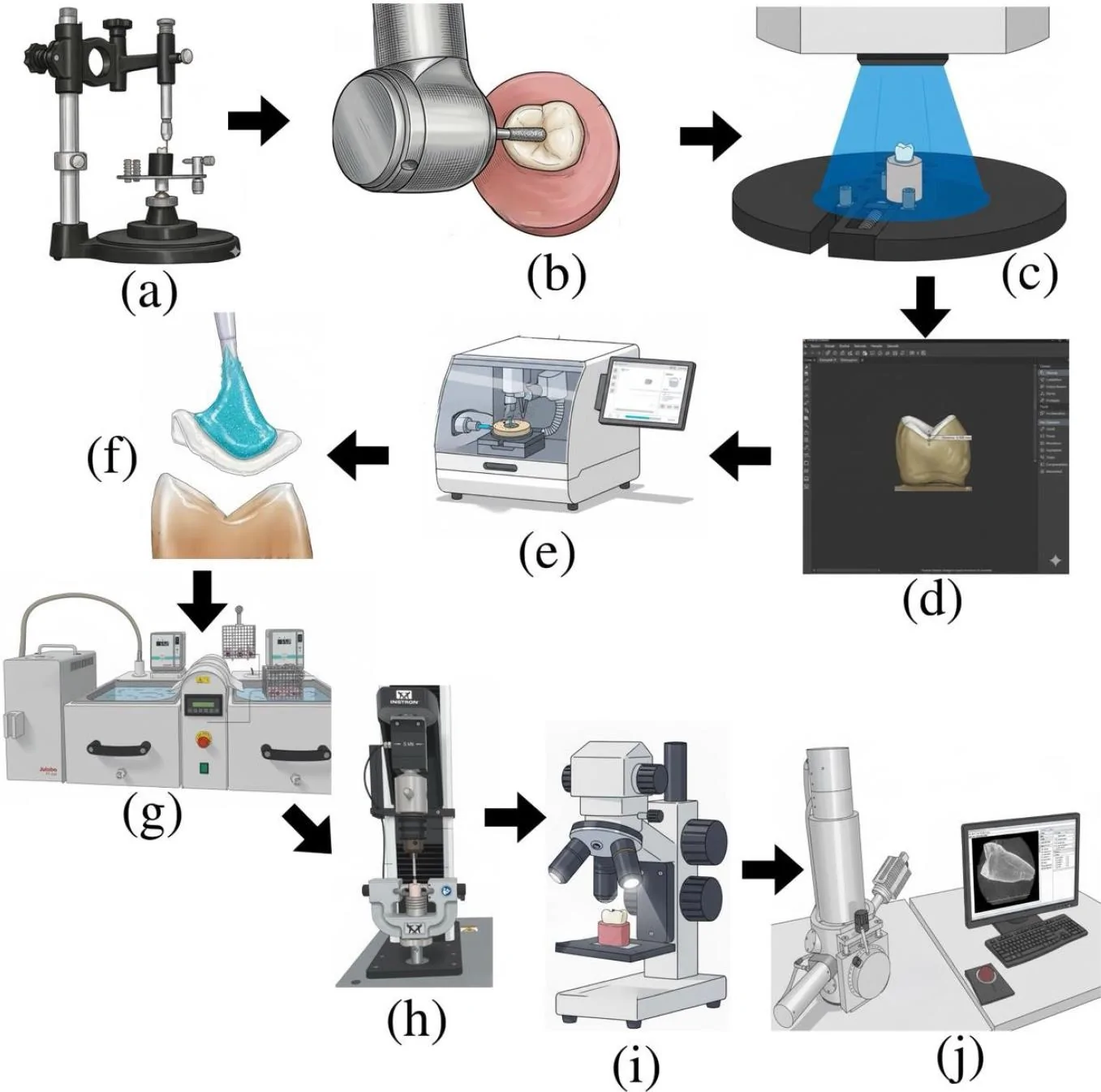
A diagram illustrating the steps of this study. (a) A parallometer apparatus used for mounting the teeth. (b) Tooth preparation. (c) Scanning prepared teeth with Medit T710 desktop scanner. (d) Designing occlusal veneers with 0.5-mm thickness. (e) Milling occlusal veneer restorations. (f) Cementation of specimens. (g) Thermocycling of specimens. (h) Fracture test using Universal Testing Machine. (i) Examination of specimens using a stereomicroscope. (j) Examination of specimens using an SEM

 Tooth preparation was done by a single experienced clinician using a magnifying dental loupe (3X). Preparation was performed exclusively within the enamel using an 830RL diamond stone (Intesiv SA, Montagnola, Switzerland) with an air-water spray at high speed. The natural inclinations of buccal and lingual cusps were maintained at 120º without a finish line. Then, preparation was finished using PrepTwinsfinishing stones (Intensiv SA, Montagnola, Switzerland) at low speed (Figure 1b). After being prepared, the teeth were inspected for cracks or other defects using a stereomicroscope.

 A digital model of the prepared teeth was created by scanning them using a desktop extraoral scanner (Medit T710, Medit Corp, Seoul, Korea) (Figure 1c). Twenty-four ultrathin occlusal veneers were made and divided into three groups of eight each: LDS (IPS e.max^®^ CAD), ZLS (Vita Ambria^®^), and ZIR (Katana^®^ UTML) (Table 1). Occlusal veneer designs were digitally created using dental CAD software (ExoCAD version 2.3, Germany). Restoration parameters were set to include a 50-μm spacer thickness and a constant occlusal thickness of 0.5 mm (Figure 1d).

**Table 1:** Materials, manufacturers, lot number, and chemical composition

**Material**	**Manufacturer**	**Lot No.**	**Chemical composition**
Lithium disilicate	Ivoclar Vivadent AG, Schaan, Liechtenstein	Z02M53	SiO_2_, Li_2_O, K_2_O, P_2_O_5_, ZrO_2_, ZnO, other coloring and stabilizing oxides
Zirconia-reinforced lithium disilicate	Vita Zahnfabrik, Bad Säckingen, Germany	82370	SiO_2_, Li_2_O, ZrO_2_, Al_2_O_3_, P_2_O_5_, K_2_O, B_2_O_3_, CeO_2_, Tb_4_O_7_, V2O5, Er_2_O_3_, Pr_6_O_11_
Zirconium dioxide	Kuraray Noritake Dental Inc., Tokyo, Japan	EIMWQ	ZrO_2_ and HfO_2_, Er_2_O_3_, Fe_2_O_3_, Y_2_O_3_, Al_2_O_3_

 Sixteen occlusal veneers were created using a CAM dental machine (VHF S5, MediMatch dental laboratory, Germany) with Katana^®^ UTML blank and IPS e.max^®^ CAD blocks (Figure 1e). Eight occlusal veneers were fabricated from a CAD-wax disc (VITA CAD-Wax; VITA Zahnfabrik) using a CAM dental milling machine (Roland DWX-510, Japan).

 LDS occlusal veneers were machined and then crystallized in a ceramic furnace (Programat EP3010, Ivoclar Vivadent, Austria) for 30 minutes at 880°C. After being polished with rubber cups and heated at 700°C, the occlusal veneers were glazed.

 In a sintering zirconia furnace (Dekema Austromat 674, Freilassing, Germany), zirconia occlusal veneers that had been machined were sintered to full density at a rate of 10°C per minute, reaching a final temperature of 1500°C.

 The milled wax templates were sprued and invested, then heated in a furnace from room temperature to 850°C at 5°C/min, with a 60-minute hold, to ensure complete wax removal. The resulting mold was subsequently heat-pressed from 700°C to 898°C at 60°C/min using pressable Vita Ambria^®^ ingots. After that, the mold was kept there for 25 minutes. Any remnants of the vesting material were removed by air abrasion at a pressure of 2 bars (50-µm aluminum oxide) after the investment was carefully removed. After polishing, the surface was glazed.

 Each specimen was ultrasonically cleaned in distilled water for 10 minutes before cementation and subsequently let to air-dry. A 9.5% hydrofluoric acid (HF) porcelain etching gel (Porcelain Etchant^®^, BISCO Inc., USA. Lot No. 2200002161) was used to etch the intaglio surfaces of the LDS and ZLS specimens for 20 seconds. This was followed by a 60-second air-water spray rinse. Prior to applying primer, the specimens were ultrasonically cleaned for 4 min in a distilled water bath and then left to air-dry. A ceramic primer containing a silane coupling agent (Porcelain Primer^®^, BISCO Inc., USA; Lot No. 2200002071) was used to prime the etched surfaces of the specimens. The intaglio surface of the ZIR specimens was abraded using 50-μm aluminum oxide particles at a pressure of 2 bars, a distance of 10 mm, and a perpendicular orientation to the surface for 15 s/cm^2^. Then, all the specimens were ultrasonically cleaned for another 4 minutes and allowed to air-dry. The intaglio surface of the ZIR group was primed with zirconia primer (Z-Prime^®^ Plus, BISCO Inc., USA; Lot No. 2300110183), then a second coat was applied and left to air-dry.

 With a 35% phosphoric acid gel (Select HV^®^ ETCH, BISCO Inc., USA; Lot No. 2100001026), the enamel was etched for 15 seconds, rinsed with an air-water spray for 30 seconds, and then left to air-dry. All-Bond Universal^®^, a dental adhesive bonding agent (Lot No. 2200005591, BISCO Inc., USA), was applied, rubbed, air-thinned, and then light-cured for 20 seconds. Dual-cured resin cement (TheraCem^®^, BISCO Inc., USA. Lot No. 2200000549) was used to cement the occlusal veneers. The restoration was carefully seated on its corresponding prepared surface (Figure 2), allowing the cement to run off all sides, after cement had been applied directly into the fitting surface of the restoration and the prepared surface. After confirming full seating, the restored teeth were first tack-cured for 2 seconds using an LED light-curing device (LED curing light, WOODPECKER, China). The excess luting material that was partially solid was then scraped off using a pointed explorer. Next, glycerin, an air-inhibiting gel, was added to the edges. Final curing lasted 30 seconds on each surface (Figure 1f).

**Figure 2:**
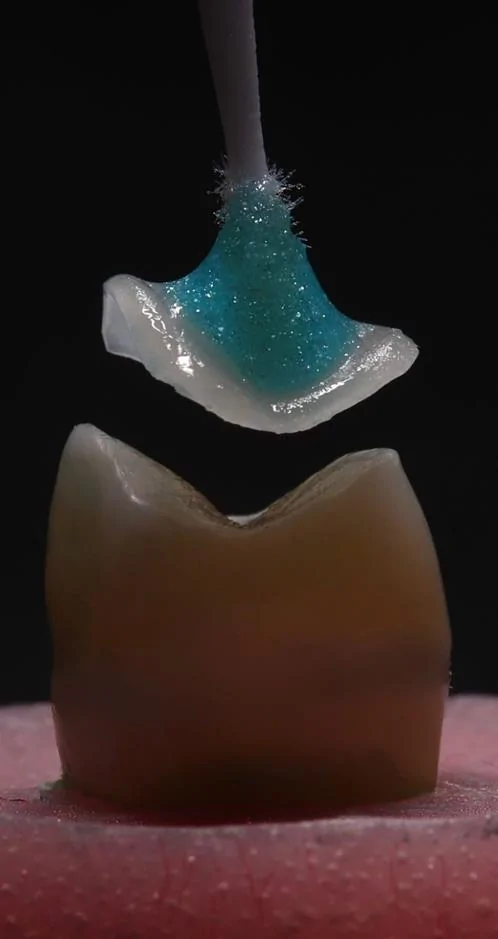
Ultrathin occlusal veneer during cementation to its corresponding tooth

 Following 24 hours of storage in distilled water, all the specimens underwent 1,200,000 thermocycling cycles (Thermocycler THE 1100, SD Mechatronics, Westerham, Germany), equivalent to about 5 years of clinical service. In accordance with the ISO 11405 guidelines, the specimens were turned between 5 and 55°C, with a 5-second dwell time and a 5-second transfer time between each bath (Figure 1g).

 All surviving specimens were placed on a universal testing machine (Model 3345; Norwood, MA, USA) equipped with a 5-kN load cell (Figure 1h). To simulate an opposing cusp, a metallic rod with a spherical tip (3.6 mm in diameter) and a cross-head speed of 0.5 mm/min was used to apply a compressive force that was directed at the fissure of the occlusal surface along the long axis of the specimens during the fracture test. A layer of tin foil with a thickness of 0.5 mm was used and adapted on the occlusal surface. The maximum force required to fracture was measured in Newtons (N). The data was recorded using BlueHill 3 software version 3.3.

 All specimens were examined with a stereomicroscope (Nikon Japan, SMZ745T Stereomicroscope, USA) and an SEM (Tescan VEGA 3, Czech Republic) to determine the mode of failure. The fracture surfaces of each specimen were first assessed and photographed using a stereomicroscope (Figure 1i), then coated with gold (Au) for 180 seconds using a Quorum Ltd. Sputter coater (Quorum Ltd, Q150T, England), and then scanned by SEM for higher-resolution photographs (Figure 1j).

 Data were statistically analyzed by Microsoft Excel^®^ 2016, SPSS^®^ Ver. 24, and Minitab^®^ statistical software Ver. 16. Qualitative data were evaluated for significance using chi-squared test, while quantitative data were revealed as minimum, median, maximum, mean, standard deviation, standard error of the mean, and the 95% confidence interval for the mean were statistically analyzed using one-way analysis of ANOVA followed by multiple comparisons using post hoc Tukey tests.

## Results

Table 2 presents the results of a one-way ANOVA, which examined statistically significant differences in the mean fracture resistance values between these groups. However, the ANOVA test does not specify which pairs of groups differ significantly. To determine this, a post hoc test, such as the Tukey test, is typically performed.

**Table 2:** Fracture resistance comparative statistics of different occlusal veneers using one-way ANOVA

	**LDS**	**ZLS**	**ZIR**	***P*** **-value**
Min	1320	1066	813.0	< 0.0001*
Max	2317	1306	995.0	
M	1741	1192	866.9	
± SD	308.3	89.44	60.23	

Min: minimum; Max: maximum; M: mean; SD: standard deviation; *P*: probability level’ *: significant difference

 The results of post hoc Tukey tests are presented in Table 3, which compares the mean differences between each pair of groups. These findings showed that the mean fracture resistance of the three tested groups varied significantly. Specifically, LDS showed the highest mean fracture resistance (1741 ± 308.3 N), followed by ZLS (1192 ± 89.44 N), and then ZIR (866.9 ± 60.23 N), which had the lowest mean fracture resistance.

**Table 3:** Multiple comparative statistics of the tested materials’ fracture resistance using post hoc Tukey tests

**Tukey's multiple comparisons test**	**95% CI of diff.**	***P*** **-value**
LDS vs. ZLS	311.3‒786.7	< 0.0001*
LDS vs. ZIR	636.1‒1111	< 0.0001*
ZLS vs. ZIR	87.07‒562.4	0.0066*

MD: mean difference; CI: confidence interval; *P*: probability level; *: significant difference

 SEM and stereomicroscope images (Figures 3 and 4) were used to investigate and evaluate the failure mode based on the AAE’s criteria, which were divided into five classes and analyzed using the chi-squared test and presented in Table 4. In the restoration debonding failure mode, the ZIR group differed significantly from the other two groups, but no significant difference was observed between the other groups. Other failure modes showed no significant differences among the three tested groups.

**Figure 3:**
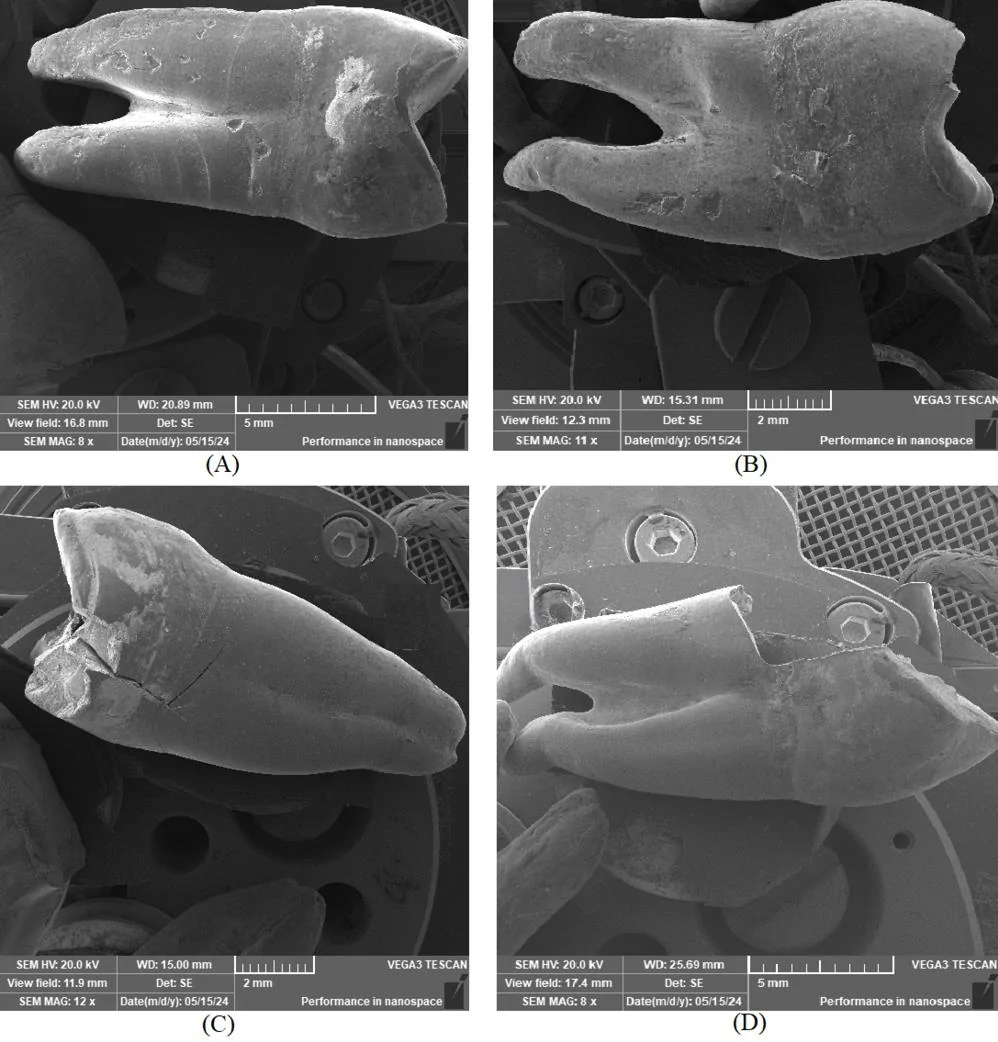
Scanning Electron Microscope images (SEM) showing different failure modes. (A): Katana® UTML debonding with repairable tooth, (B): IPS e.max® CAD fracture with repairable tooth, (C): Vita Ambria® fracture with irreparable tooth, (D): Katana® UTML fracture with irreparable tooth

**Figure 4:**
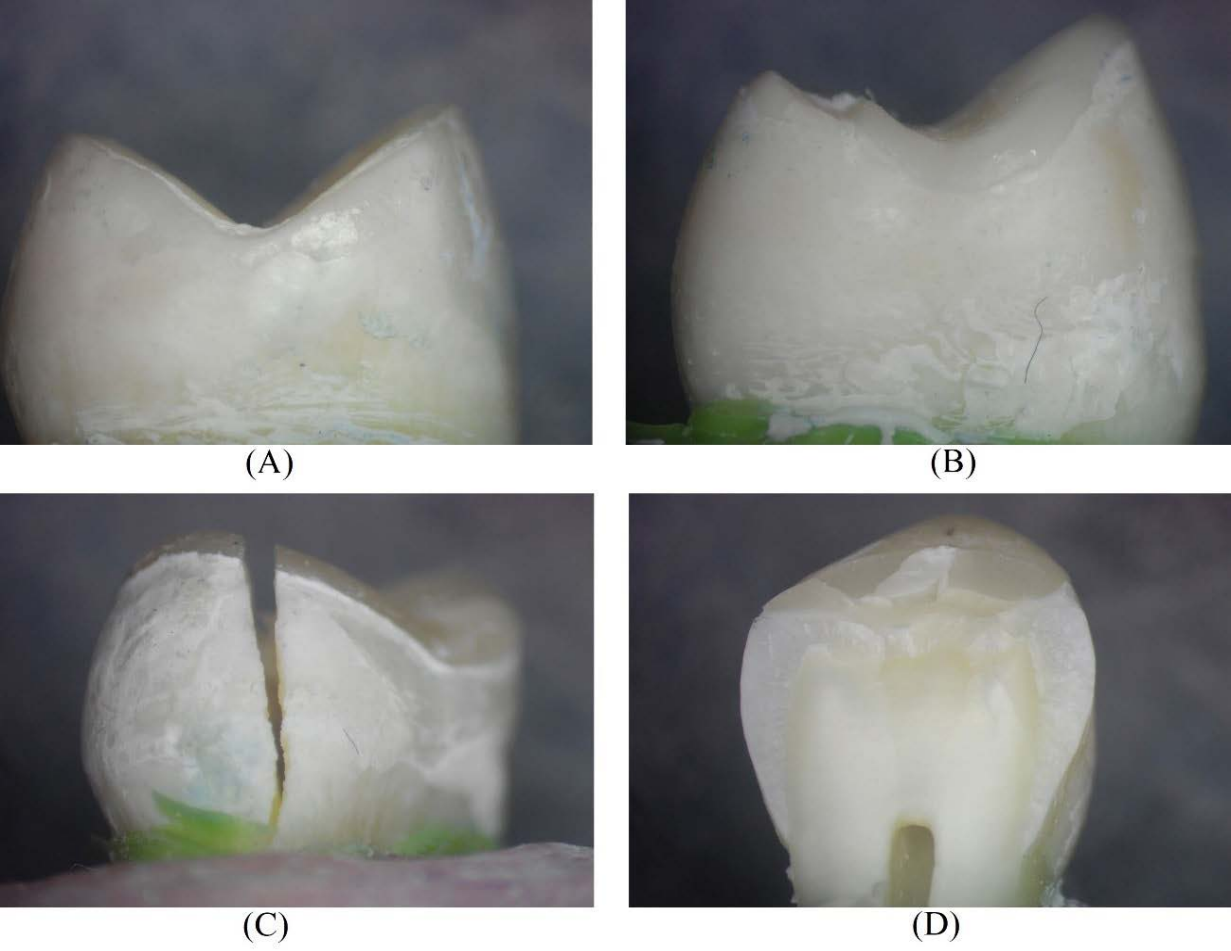
Stereomicroscope images showing different failure modes. (A): Katana® UTML debonding with repairable tooth, (B): IPS e.max® CAD fracture with repairable tooth, (C): Vita Ambria® fracture with irreparable tooth, (D): Katana® UTML fracture with irreparable tooth

**Table 4:** Comparative statistics of failure mode percentage of the three tested materials using the chi-squared test

**Mode of Failure**		**LDS**		**ZLS**		**ZIR**		***P*** **-value**
		**N**	**%**	**N**	**%**	**N**	**%**	
Restoration	Restoration deformation with unaffected tooth	0	0%	0	0%	0	0%	1.0000
	Restoration fracture with an unaffected tooth	1	12.5%	2	25%	1	12.5%	0.5351
	Restoration debonding	0	0%	0	0%	4	50%	0.0253*
Tooth	Craze line	0	0%	0	0%	0	0%	1.0000
	Fractured cusp	5	62.5%	5	62.5%	2	25%	0.1432
	Cracked tooth	0	0%	1	12.5%	0	0%	0.3173
	Split tooth	2	25%	0	0%	1	12.5%	0.5351
	Vertical root fracture	0	0%	0	0%	0	0%	1.0000

N: number; %: percentage; *P*: probability level; *: significant difference

 Frequencies and percentages of the reparability of restored teeth were analyzed using the chi-squared test and presented in Table 5. There is no statistically significant difference among the three materials tested.

**Table 5:** Comparative statistics of the repairability percentage of the three tested materials using Chi Square test

**Repairability**	**LDS**		**ZLS**		**ZIR**		***P*** **-value**
	**N**	**%**	**N**	**%**	**N**	**%**	
Repairable fracture	6	75%	6	75%	7	87.5%	.777 (NS)
Irreparable fracture	2	25%	2	25%	1	12.5%	

N: number; %: percentage; *P*: probability level; Ns: nonsignificant difference

## Discussion

 Occlusal veneers have evolved to preserve tooth structure and achieve optimal adhesion, with recent advances in adhesive bonding. Anatomical considerations and interocclusal clearance are the main factors in determining the simple tooth preparation needed for partial-coverage restorations. Their indications have grown to include the treatment of oral rehabilitation situations, such as lost functional occlusion, and cases of severe attrition and advanced erosion caused by bruxism.^16^

 The fracture strength of ceramic restorations is considered a challenge influenced by several parameters, including preparation design, substructure, luting cement, the mechanical properties of the material used, fabrication method, and occlusal functional loading.^4,10,17,18^

 The current study’s findings indicate that different restorative materials significantly affected the ultrathin occlusal veneer’s fracture resistance. Therefore, the first null hypothesis, which stated that different restorative materials would not affect the fracture resistance of ultrathin occlusal veneers, was rejected.

 There was a statistically significant difference in the fracture resistance of LDS, ZLS, and ZIR ultrathin occlusal veneers. The LDS group had the highest statistically significant mean fracture resistance, followed by ZLS and ZIR, which had the lowest. These findings may be attributed to the smaller elastic modulus mismatch between LDS (95 GPa), and the substrate enamel (85 GPa) as compared to ZLS (100 GPa) and ZIR (210 GPa). Ceramics with a modulus of elasticity that is close to the substrate exhibit enhanced stress distribution and load-bearing capacity. Furthermore, the strength of the tooth‒restoration complex increases when the substrate and restorative material have similar elastic moduli.^5,19^ The fracture resistance of ceramic restorations that are uniformly supported and bonded is influenced by the difference in elastic moduli between the ceramic and the substrate. Even though the effect of elastic modulus mismatch on fracture resistance is less significant than that of ceramic strength, a lower mismatch can significantly increase the load-bearing capacity of ceramic restorations.^20^

 Moreover, the adhesive luting technique, which allows for intimate contact between the enamel, luting agent, and etchable and adhesively bondable ceramics such as LDS and ZLS, may be related to these results. Because of such intimate contact, occlusal stresses can be transmitted through the tooth, periodontal ligament, and alveolar bone.^21^ Furthermore, the integration of an optimal adhesive bonding system considerably strengthens weak and thin restorations when firmly bonded to enamel, causing them to behave better than strong or thick restorations. Conversely, non-etchable ceramics, such as ZIR, had difficulty adhering to the tooth structure, potentially reducing adhesive bonding compared with etchable ceramics.^10^

 LDS ceramics are homogeneous materials and have few structural defects. This property is crucial and may have played a part in the high fracture load values of the ultrathin occlusal veneers, as more homogeneous materials experience less internal stress, which increases the material’s fracture load in comparison to other less homogeneous materials.^5^ Furthermore, LDS has a monolithic structural feature, which facilitates HF etching and strengthens the bond with the adhesive resin cement.^10^

 Al-Akhali et al.^10^ examined the fracture resistance of minimally invasive occlusal veneer restorations on human maxillary premolars after thermomechanical fatigue loading and found that LDS exhibited a statistically significant difference relative to ZLS, which is in agreement with these findings. Additionally, Zumstein et al.^11^ investigated the load-bearing capability of various pressable lithium disilicates cemented as occlusal veneers on molars; they found that pressable LDS showed a statistically significant difference compared with ZLS. It has been demonstrated that adding zirconia to a lithium disilicate ingot did not improve its mechanical properties. According to flexural tests, zirconia additions exceeding 10% decreased flexural strength.^22^

 In contrast to the current study’s findings, Maeder et al.^9^ reported that ZIR occlusal veneers achieved greater fracture resistance than LDS when applied on molars at a thickness of 0.5 mm. Al-Zordk et al.^6^ tested ZIR and LDS as occlusal veneers on molars with a thickness of 1 mm and found no discernible differences between them. The use of molar teeth with greater preparation angles and of various substrates with varying moduli of elasticity could be the cause of this discrepancy.

 Based on the current study’s findings, the second part of the null hypothesis—that there would be no significant difference in the modes of failure of the three tested materials—was partially rejected. Since the ZIR group showed a statistically significant difference in restoration debonding compared to the other two groups.

 Compared with the other two ceramics examined, ZIR restorations had a lower catastrophic failure rate. Zirconia is difficult to bond to tooth structure; therefore, when a restoration is loaded, there is a greater chance to debond from the tooth than it will bear the stress to the point of fracture.^23^ Moreover, the ZIR group was the only one to exhibit the restoration debonding failure mode. It was found that the most common long-term failure of zirconia restorations was debonding. In a previous study, Tribst et al.^24^ assessed stresses in occlusal veneers by restoration type and found that ZIR had the highest stress concentration, followed by LDS and polymer-infiltrated ceramics (PIC). Additionally, they discovered that PIC caused the most stress on the cement layer, followed by ZIR and LDS.

 Regarding tooth repairability after fracture of specimens, 87.5% of the ZIR group samples had repairable fractures. This may be due to restoration debonding, the main mode of failure, which occurs in 50% of specimens. Nonetheless, repairable tooth fractures were present in 75% of the LDS and ZLS group samples. This could be explained by their modulus of elasticity, which affects a ceramic restoration’s susceptibility to fracture, as materials with a modulus of elasticity comparable to the underlying substrate bend and distribute stresses more uniformly under load. Nevertheless, materials with different elastic moduli cause stress concentrations at critical points, which may lead to catastrophic failures.^4^

 The most common failure mode observed in the current study was fracture involving both the tooth and the restoration. This implies that the restorations have strength comparable to the damaged teeth they are meant to replace. The tooth‒restoration complex is favored when the substrate and restorative material have a comparable elastic modulus.^6,19^ Additionally, with glass ceramics, adhesive bonding to the tooth structure allows for all force transmission via the restoration‒tooth complex.^6^ However, the applied stresses can cause fractures to form and spread, which could lead to structural collapse and fractures that could damage the tooth structure.^25^

 In the current study, extracted maxillary first premolars were selected because when premolars are prepared for a full-coverage crown, their distinct anatomic shape—narrow in the cervical third of the crown relative to the occlusal surface—usually results in an excessive loss of tooth structure.^26^ Natural teeth were selected due to their superior clinical simulation and bonding qualities, heat conductivity, modulus of elasticity, and strength compared to metal dies, epoxy resins, or bovine teeth. The teeth were approximately the same size and dimension to ensure standardization of the occlusal veneer restorations, with similar elastic moduli.

 Preparations were performed exclusively within the enamel to enhance the adhesive cementation of occlusal veneers. It has been demonstrated that minimally invasive ceramic restorations that are only bonded to enamel have a notably higher level of fracture resistance in comparison to those that are bonded to dentin.^19^ Moreover, some previous investigations found that adhesively bonded thin restorations to enamel exhibited a high level of fracture resistance that was on comparable levels with thick restorations bonded to dentin.^27,28^ It is also essential to evaluate the preparation method to maintain the tooth structure and improve stress distribution during the fracture load test.^29^

 The preparation was limited to the occlusal surface and did not include a finish line at the axial wall since it was discovered that neither the presence of a finish line nor the preparation design in dentin or enamel influenced the occlusal veneers’ fracture resistance.^27^ When a thin occlusal veneer was made with a finish line along the axial walls, stress concentration areas formed at the cusp points underneath it.^15,30,31^ Furthermore, it was demonstrated that, in comparison to conventional crowns and veneers that partially covered the axial surfaces, occlusal veneers that covered the occlusal surface showed a reduction in the stresses created in the restoration and increased fracture resistance.^6,26^

 Long-lasting chemical and micromechanical resin bonding in zirconia is not possible with conventional methods such as HF acid etching and silane coupling agents. To strengthen the zirconia‒resin bond, a variety of mechanical, chemical, and mixed chemomechanical surface treatment techniques have been available. One of these pretreatment techniques—airborne particle abrasion—aims to strengthen mechanical and chemical bonds.^32^ Surface treatment with primers and resin cements based on 10-methacryloyloxydecyl dihydrogen phosphate (MDP) monomer or phosphate ester monomer produces superior and durable bonding between zirconia and resin. These functional monomers establish stable chemical bonds with metal oxides present in zirconia.^33^ Z-Prime^®^ Plus, which was used for ZIR, has two adhesive monomers: carboxylic acid monomer and organophosphate monomer. However, it was shown that greater bond strengths to zirconia were obtained when priming agents containing (MDP) were used instead of those containing carboxylic monomer.^6,34^

 Five years of clinical service were replicated by thermocycling all specimens at 1,200,000 cycles.^4^ The bond strength is affected by temperature variations during thermocycling. In the literature, there is ongoing debate about the extent to which thermocycling affects bond strength. It ought to be a part of the artificial aging process, since zirconium dioxide restorations spontaneously lose their adhesive bond after thermocycling.^35^

 Clinically, the maxillary premolar region’s normal biting force is 222‒445 N, while the occlusal force during clenching is 520‒800 N.^26^ The mean fracture load values in the current study were higher than those values, indicating that all materials examined can withstand the maximal bite force without fracturing. However, as the applied force range in this study was limited and people’s masticatory forces and habits vary widely, it should be deemed premature to reach this conclusion. Because this study examined only static strength, it was unable to replicate all aspects of occlusal loading or the long-term effects of the oral environment on the restoration’s strength.

 Disparities in the techniques employed in previous studies, such as adhesive luting procedures, mechanical loading, restoration thickness, preparation, and substrates, may account for the diversity of results. Fracture resistance values in this study cannot be directly compared with those reported in other related studies because premolars were used in this investigation. One of the study’s limitations is that the complexity of the oral environment and masticatory biomechanics cannot be reproduced in vitro. While the occlusal surfaces of worn teeth are covered in sclerotic dentin and may have varying adhesive bonding, teeth were formed entirely within enamel. Testing multiple thicknesses, alternate designs, resin cement types, and dental bonding substrates is required.

## Conclusion

 Ultrathin occlusal veneers showed fracture resistance values that exceeded the physiologic functional occlusal loads. Compared with the ZLS and ZIR groups, LDS had the highest fracture resistance. ZIR had a higher chance of repairability than LDS and ZLS.

## Competing Interests

 None.

## Consent for Publication

 No consent is required for this in vitro study.

## Ethical Approval

 Ethical approval (No. 823/3071) was granted by the Ethics Committee of the Faculty of Dental Medicine (Boys), Al-Azhar University, Cairo, Egypt.
